# Regulation of mechanical stress by mammary epithelial tissue structure controls breast cancer cell invasion

**DOI:** 10.18632/oncotarget.979

**Published:** 2013-04-12

**Authors:** Derek C. Radisky, Celeste M. Nelson

**Affiliations:** Department of Cancer Biology, Mayo Clinic Cancer Center, Jacksonville, FL; Departments of Chemical and Biological Engineering and Molecular Biology, Princeton University, Princeton, NJ

The behavior of cells within tissues is controlled by interactions with soluble signals, neighboring cells, and the surrounding extracellular matrix (ECM); collectively, these interactions constitute the cellular microenvironment. While normal tissue homeostasis can suppress outgrowth of cells with oncogenic mutations, progression to malignant cancer is associated with alterations in the cellular microenvironment that facilitate cancer cell proliferation, detachment from adjacent cells, and penetration of the surrounding ECM [1]. Several recent studies have provided new insight into how microenvironmental signals combine to facilitate tissue invasion of individual cancer cells [2, 3]. In the first, careful monitoring of the invasion process through time-lapse microscopy was used to evaluate the behavior of normal and cancerous breast tissue explants cultured in three-dimensional (3D) ECM [3]. These studies revealed that sustained cellular invasion occurred only when cells were cultured in ECM composed of stromal collagen, and also that acquisition of the invasive phenotype was relatively rare, occurring only in a very small fraction of the cells in the explant. Furthermore, invasion was found to occur preferentially at regions that protruded from the tissue fragments, and was preceded by loss of cell-cell adhesions [3]. These studies indicated that cellular invasion required a combination of the correct tissue structural orientation as well as specific cell-cell and cell-ECM interactions.

To define how cell-cell and cell-ECM interactions are integrated in a tissue context, Boghaert et al. [2] used microengineered tissues, created by embedding single breast cancer cells within a surrogate duct structure composed of nonmalignant mammary epithelial cells in 3D collagen molds (Figure 1A,B). This model system was used to parse out the tissue-based biophysical characteristics that control cancer cell invasion. When individual invasive cancer cells were incorporated into tissue surrogates composed of nonmalignant cells, invasion occurred preferentially at the protrusive ends of tissue structures (Figure 1C). Computational modeling and experimental microbead displacement studies revealed that these regions showed the highest endogenous mechanical stress; investigation of different tissue morphologies and orientations revealed that tissue stress was induced by contraction of the surrogate ducts within 3D collagenous matrix (Figure 1D-F). Reduction of mechanical stress in the entire tissue structures by treatment with inhibitors of actomyosin contractility inhibited tumor cell invasion from the tissue ends, demonstrating that contraction is required for invasiveness. However, inhibiting either actomyosin contractility or intracellular transmission of mechanical stress *specifically* in the normal host epithelial cells allowed embedded tumor cells to invade from all locations within the tissue structure. This demonstrates that control of malignant cell behavior by the normal tissue depends upon structural integrity of the tissue. These findings were evaluated in a more complex 3D model of mouse mammary gland tissue morphology that was generated by microcomputed tomography, with subsequent computational modeling of endogenous contractility in the ductal epithelial structure (Figure 1G-J). These studies predicted significantly elevated mechanical stress at the ends of the epithelial tree as compared with the shafts of the ducts, and were consistent with patterns of tumor formation in transgenic mouse breast cancer models.

**Figure 1:**
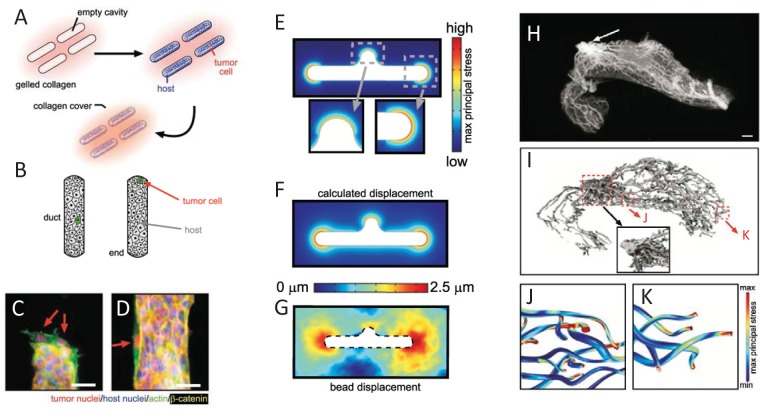
Epithelial tissue morphology controls mechanical stress A. Schematic of 3D microlithography-based approach for engineering epithelial tissue structures. B. Schematic depicting duct vs end locations in engineered epithelial tissues. C-D. Tumor cells (red nuclei) invading from end of tissue (C) and suppressed from invading from the duct (D). E. Predicted endogenous mechanical stress of tissue structure. F. Predicted displacement of tissue structure. G. Experimentally measured bead displacement assessed in culture. H. Microcomputed tomography volume rendering of inguinal mammary gland of 8-week mouse. I. 3D rendering of the network of epithelial ducts. (*Inset*) Detailed view of ductal network near nipple. J-K. Computational model of maximum principle stress along the ducts (J) and at the ends (K) of the epithelial network. Material reprinted from reference 2.

It is now becoming clear that the mechanical properties of the cell are critically important for regulation of a variety of cellular functions, including cytokinesis and locomotion [4], although how these processes are dynamically regulated in epithelial tissues by interaction with neighboring cells and with the ECM has been a more difficult process to study. Much has been learned from use of 3D model systems with tissue explants and cultured cancer cells [5], and extension of these studies using tissue engineered surrogate ducts now provides a powerful method to dissect how biochemical and biomechanical signals are integrated by the host tissue for control of normal morphogenesis as well as cancer invasion and progression. An important extension of this approach will be to integrate the effect of higher macromolecular ECM structures in the invasion/morphogenic processes; for example, bundling of collagen into fibrils, the presence and orientation of which can control tissue response [6]. Additionally, just as the epithelial tissue changes during tumor development, so does the structure and biomechanical properties of the surrounding stroma [7], as well as the abundance and composition of different cell types within the stroma, which play a critical role in tumor progression [8].

## References

[1] Bissell M.J, Hines W.C (2011). Why don't we get more cancer? A proposed role of the microenvironment in restraining cancer progression. Nat Med.

[2] Boghaert E. (2012). Host epithelial geometry regulates breast cancer cell invasiveness. Proc Natl Acad Sci U S A.

[3] Nguyen-Ngoc K.V (2012). ECM microenvironment regulates collective migration and local dissemination in normal and malignant mammary epithelium. Proc Natl Acad Sci U S A.

[4] Tee S.Y, Bausch A.R, Janmey P.A (2009). The mechanical cell. Curr Biol.

[5] Yamada K.M, Cukierman E (2007). Modeling tissue morphogenesis and cancer in 3D. Cell.

[6] Brownfield D.G (2013). Patterned Collagen Fibers Orient Branching Mammary Epithelium through Distinct Signaling Modules. Curr Biol.

[7] Plodinec M (2012). The nanomechanical signature of breast cancer. Nat Nanotechnol.

[8] Cichon M.A (2010). Microenvironmental influences that drive progression from benign breast disease to invasive breast cancer. J Mammary Gland Biol Neoplasia.

